# Electrostatic-Fluid-Structure 3D Numerical Simulation of a MEMS Electrostatic Comb Resonator

**DOI:** 10.3390/s22031056

**Published:** 2022-01-29

**Authors:** Zhanqing Yu, Shiping Chen, Ya Mou, Fade Hu

**Affiliations:** 1 State Key Lab of Power Systems, Department of Electrical Engineering, Tsinghua University, Beijing 100084, China; csp20@mails.tsinghua.edu.cn (S.C.); mouy17@mails.tsinghua.edu.cn (Y.M.); 2 Department of Precision Instrument, Tsinghua University, Beijing 100084, China; hfd20@mails.tsinghua.edu.cn

**Keywords:** MEMS, microelectrostatic comb resonator, multifield coupling, dynamics research, air damping, finite element analysis

## Abstract

The reliability and stability of MEMS electrostatic comb resonators have become bottlenecks in practical applications. However, there are few studies that comprehensively consider the nonlinear dynamic behavior characteristics of MEMS systems and devices in a coupled field so that the related simulation accuracy is low and cannot meet the needs of design applications. In this paper, to avoid the computational complexity and the uncertainty of the results of three-field direct coupling and take into the damping nonlinearity caused by coupled fields, a novel electrostatic-fluid-structure three-field indirect coupling method is proposed. Taking an actual microcomb resonant electric field sensor as an example, an electrostatic-fluid-structure multiphysics coupling 3D finite element simulation model is established. After considering the influence of nonlinear damping concerning the large displacement of the structure and the microscale effect, multifield coupling dynamics research is carried out using COMSOL software. The multiorder eigenmodes, resonant frequency, vibration amplitude, and the distribution of fluid load of the microresonator are calculated and analyzed. The simulated data of resonance frequency and displacement amplitude are compared with the measured data. The results show that the fluid load distribution of the microelectrostatic comb resonator along the thickness direction is high in the middle and low on both sides. The viscous damping of the sensor under atmospheric pressure is mainly composed of the incompressible flow damping of the comb teeth, which is an order of magnitude larger than those of other parts. Compared with the measured data, it can be concluded that the amplitude and resonance frequency of the microresonator considering the nonlinear damping force and residual thermal stress are close to the experimental values (amplitude error: 15.47%, resonance frequency error: 12.48%). This article provides a reference for studies on the dynamic characteristics of electrostatically driven MEMS devices.

## 1. Introduction

With the development of micro/nanoprocessing technology and Internet of Things technology, microelectromechanical systems (MEMS) are being widely used in electronic communications, smart homes, wearable electronic equipment, medical care, and transparent power grids. MEMSs have the characteristics of miniaturization, high integration, and suitability for low-cost mass manufacturing. Among them, MEMS resonators occupy a large part of the market applications, including sensing [1,2,3,4], timing [5,6], and radio frequency communication [7,8]. Electrostatic comb drive structures have become one of the most important driving methods in MEMSs due to their advantages of low power consumption and high speed. However, the reliability and stability of microcomb resonators have become bottlenecks in practical applications, restricting the development and market access of related MEMS products. In an actual working environment, a microresonator is subject to interactions with multiple physical fields, including electrostatic fields, fluid fields, and temperature fields. There are few studies on the multifield coupling characteristics and the nonlinear dynamic behavior characteristics of MEMS systems, including nonlinear effects on the response of resonant MEMS devices [9], collective behaviors of mechanically and electrically coupled M/NEMS resonators [10], and geometric and electrostatic nonlinearities of double-ended tuning fork MEMS resonators [11]. Therefore, to accurately predict the characteristic parameters of a MEMS resonator, including the resonance frequency, amplitude, and resonant mode, it is of great significance to study the multifield coupling nonlinear dynamics of microcomb resonators and to build a multifield coupling numerical model. The main goal of this paper is to improve the simulation accuracy so that it can meet the application requirements.

The dynamic research of MEMS comb resonators comprehensively applies theoretical knowledge in the fields of electrostatic fields, structural mechanics, fluid mechanics, and thermodynamics. The analysis method mainly covers the traditional analytical method, equivalent circuit representation method, and grid discrete method represented by the finite element method. The traditional analytical method generally uses the electrostatic field analytical method to solve the electrostatic driving force, applies microfluid mechanics to calculate the air damping, utilizes the Lagrange equation of the cantilever to obtain the equivalent mechanical stiffness, and then employs the dynamic control equation of the spring-mass-damping system to analyze the resonator [12], solve the natural frequency and movement displacement [13] and study the influencing factors of resonant frequency and spring stiffness [14] of the resonator. The equivalent circuit representation of lumped parameter systems [15] and distributed parameter systems [16] refers to the use of the analogy between electrical resonators and mechanical resonators to build an equivalent circuit of micromechanical resonators for rapid system design and simulation [17,18]. Although the physical concepts of the above methods are clear, they cannot comprehensively consider the large displacement of the structure, distributed mass and stiffness, multiorder vibration eigenmodes, microscale effects, residual prestress, nonlinear vibration characteristics, stiffness softening and hardening effects, energy dissipation, complex external environmental influences (temperature, humidity, pressure, etc.), multifield coupling effects and other factors, which result in limitations in accurately calculating the key performance parameters of the MEMS resonator under the multifield interaction of the electrostatic fluid structure. Some studies have used experimental curve fitting to study the parametric resonance [19], frequency response curve, stiffness hardening, and softening effects [20] of microresonators, which can achieve accurate modeling, but there are certain difficulties in wide application due to the limitations of experimental conditions.

To overcome the limitations of the analytical method, domestic and foreign studies have mostly focused on the finite element analysis method. Zhang et al. used finite element tools to extract the nonlinear stiffness of the spring [21]. Ahmed et al. studied the influence of the design parameters of the transverse electrostatic comb driver (comb tooth gap, comb tooth thickness, etc.) on the performance of the driver with the direct coupling finite element method [22]. In addition, the finite element method was applied to static, modal, and harmonic response simulation analyses of double-free beam resonators [23] and laterally vibrating microresonators [24,25].

The above theoretical studies on microelectrostatic comb resonators have been mostly carried out through electrostatic-structure bidirectional coupling simulations, without considering air damping or taking air damping as a fixed constant, to simulate the resonance frequency and amplitude of the microresonator. Due to the high-speed resonant motion of the microresonator, the air flow field distribution is different at each moment, and the air damping force on the surface of the microresonator can change at any time. Therefore, simply using a fixed damping coefficient leads to low simulation accuracy, which cannot meet the needs of design applications. However, the convergence of a three-field directly coupling finite element time-domain model is extremely low, and the calculation time is enormous. To date, there is no existing general simulation software that can realize electrostatic-fluid-structure three-field direct coupling Thence, there are few 3D electrostatic-fluid-structure multiphysics coupling finite element simulation models of microelectrostatic comb resonators that consider dynamic damping at present.

In this paper, a novel electrostatic-fluid-structure three-field indirect coupling method is proposed to avoid computational complexity and uncertainty of the results of the three-field direct coupling. This paper takes an actual microcomb resonant electric field sensor as the prototype, establishes a 3D electrostatic-fluid-structure multiphysics coupling finite element simulation model using COMSOL software, and obtains the multiorder resonance mode, resonance frequency, vibration amplitude, fluid load distribution of the microresonator. In comparison with other models reported in the literature, the proposed simulation model considers nonlinear damping concerning the large displacement of the structure and the microscale effect. And it can perform more accurate quantitative calculation and analysis of the key performance parameters of the nonlinear dynamic vibration of the MEMS resonator in the electrostatic-fluid-structure coupling, better revealing the distribution and change law of various fluid mechanics and solid mechanics quantities when the device is working. In addition, design of microresonators under uncertainties [26,27] induced by process and environmental factors will be also improved. The simulation accuracy is higher, but more simulation time is required.

## 2. Electrostatic-Structure-Fluid Coupling Model of a Microcomb Resonant Electric Field Sensor

**Figure 1:**
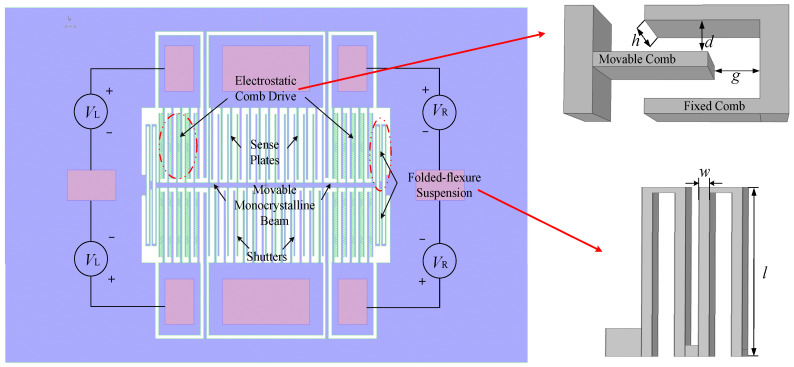
Schematic diagram of the microelectrostatic resonance electric field sensor. The purple structure is the physical structure fabricated from the top silicon film. The pink structures are sputtered metal electrodes. The dimensional parameters shown in the figure are listed in Table 1.

Taking an actual microcomb resonant electric field sensor as the prototype, a 3D dynamic simulation model is established, as shown in Figure 1. It is a typical structure of laterally vibrating microresonators that have electrostatic control. The basic structure and simulation modeling parameters that correspond to Figure 1 are shown in Table 1.

The microcomb electric field sensor introduced in this article uses silicon-on-insulator technology. The main process flow includes sputter coating of metal electrodes, etching of the front structure, etching of the back substrate, and gaseous release of the oxide layer to form a suspended and hollowed-out structure. When the microresonator is operating, the movable structure (including the folded-flexure suspension, shutters, and the movable beam and combs) applies a DC bias voltage, and the fixed end of the comb applies an AC driving voltage, which generates a periodic electrostatic force between the movable comb teeth and the fixed comb teeth, resulting in the horizontal vibration of the movable structure under the combined action of the electrostatic force and the elastic restoring force of the folded-flexure suspension. The electromechanical coupling between the movable comb teeth and the fixed comb teeth is used as the electromechanical energy conversion module of the entire resonator structure, which converts the input voltage signal into a mechanical vibration signal. Thus, the resonance frequency modulates the signal to be measured to achieve sensing. In dynamic research on microelectrostatic comb resonators, three main aspects, electrostatic mechanics, aerodynamics, and structural dynamics, are considered.

### 2.1. Electrostatic-Fluid-Structure Three-Field Coupling Process

The air damping of the movable structure is dependent on movement displacement. To increase the accuracy of the calculation, this model considers electrostatic-fluid-structure multiphysics coupling. To date, there is no existing general simulation software that can realize electrostatic-fluid-structure three-field direct coupling, which has problems such as a long calculation period and nonconvergence of calculation results. Therefore, a calculation of indirect coupling is carried out, which is decomposed into the fluid-structure coupling and electromechanical coupling. In the fluid-structure coupling time-domain transient analysis, the function of the damping coefficient with the movement displacement (*x*) is extracted as c(x).

However, the overall electric field sensor needs to be analyzed in the frequency domain of the electromechanical coupling model, where only the displacement amplitude and phase are considered. Therefore, it is necessary to convert c(x) from the concept of the time domain to the frequency domain, that is, $c_{e}(X)$, where *X* is the amplitude of vibration, with the conversion basis that the energy lost by air damping in the unit resonance period is equal:

(1) $$\int_{0}^{T}c(x)v^{2}dt=\int_{0}^{T}c_{e}(X)v^{2}dt$$

where v is the velocity of the comb teeth, T is the vibration period and the damping force is $f_{damp}=cv$.

Suppose the movement displacement of the movable part is:

(2) x(t)=Xsin(ωt+θ)=Xsinφ

Its movement speed is:

(3) $$v(t)=\dot{x}=X\omega \cos\varphi .$$

Substituting Formula (3) into Formula (1), we can obtain:

(4) $$\int_{0}^{T}c(x)\cdot {\left(X\omega \cos\omega t\right)}^{2}dt=\int_{0}^{T}c_{e}(X)\cdot {\left(X\omega \cos\omega t\right)}^{2}dt.$$

Since $c_{e}(X)$ is independent of time, it is easy to obtain:

(5) $$c_{e}(X)=\frac{2}{T}\int_{0}^{T}c(x)\cdot (\cos\omega t{)}^{2}dt.$$

**Figure 2:**
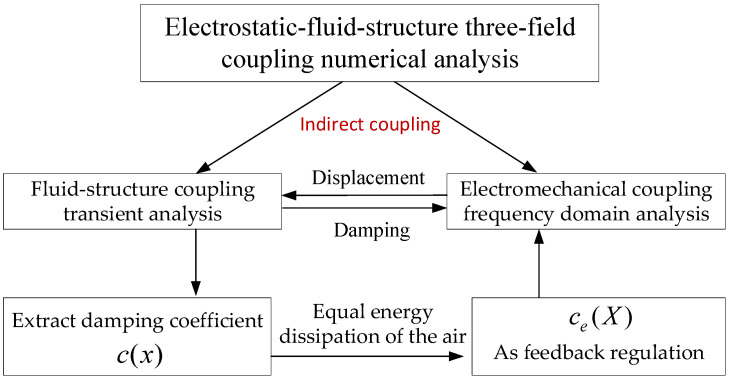
Schematic diagram of three-field coupling data transfer.

The damping coefficient as a function of the resonance amplitude (*X*) is used as the boundary condition of the electromechanical coupling frequency domain analysis, achieving two-way indirect coupling by repeated iterations. The data transfer of multiphysics coupling is shown in Figure 2.

To understand the error caused by the simplification, we inversely derived the vibration equation and found that the essence of using the energy conservation of air damping loss to perform damping time-frequency domain conversion is to perform equivalent linearization of nonlinear vibration. The simplification error comes from omitting the higher-order terms of a Fourier expansion. To facilitate understanding, the forward derivation is carried out as follows:

The nonlinear vibration equation can be expressed in the following form:

(6) $$m\ddot{x}+c(x)\dot{x}+kx=F\sin\omega t.$$

where m is the effective mass, x is the movement displacement, k is the mechanical equivalent stiffness, and F is the magnitude of the applied electrostatic force.

The equivalent linearized vibration equation corresponding to it is:

(7) $$m\ddot{x}+c_{e}\dot{x}+kx=F\sin\omega t.$$

where $c_{e}$ is the equivalent damping coefficient. The nonlinear damping force is expanded according to the Fourier series:

(8) $$c(x)\dot{x}=a_{0}+\sum_{n=1}^{\infty }\left(a_{n}\cos n\varphi +b_{n}\sin n\varphi \right),n=1,2,3\ldots$$

Since only the first harmonic force of the Fourier series is kept, Equation (13) can be approximated as:

(9) $$c(x)\dot{x}=a_{0}+a_{1}\cos\varphi +b_{1}\sin\varphi .$$

By substituting Formula (9) into Formula (6), we can obtain:

(10) $$m\ddot{x}+\left[\frac{1}{\pi }\int_{0}^{2\pi }c(X\sin\varphi )\cos^{2}\varphi d\varphi \right]\dot{x}+kx=F\sin\omega t$$

The equivalent damping coefficient $c_{e}$ is

(11) $$c_{e}=\frac{1}{\pi }\int_{0}^{2\pi }c(X\sin\varphi )\cos^{2}\varphi d\varphi =\frac{2}{T}\int_{0}^{T}c(x)\cdot (\cos\varphi {)}^{2}dt$$

Formula (11) is exactly equal to Formula (5).

### 2.2. Fluid–Solid Coupling Calculation Model

**Figure 3:**
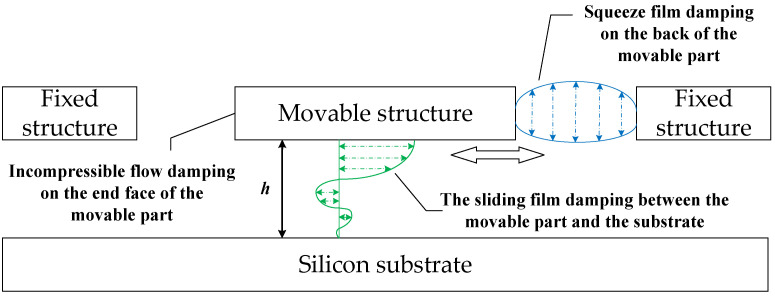
Side view of viscous damping distribution of microresonator.

Viscous damping is a significant force in MEMS devices operated at atmospheric pressure, particularly at resonance. Therefore, support loss, thermoelastic damping, and material damping are neglected. Due to the scale effect of the microstructure, air damping is an important factor affecting its dynamic characteristics. It determines the quality factor of the resonator, the displacement amplitude of the shutter, the accuracy and stability of the system, and other key parameters. The viscous damping of the microcomb mechanical resonator mainly includes slide film damping between the movable part of the resonator surface and the base and side air damping (including squeeze film damping and incompressible flow damping), as shown in Figure 3.

#### 2.2.1. Slide Film Damping and Squeeze Film Damping

**Figure 4:**
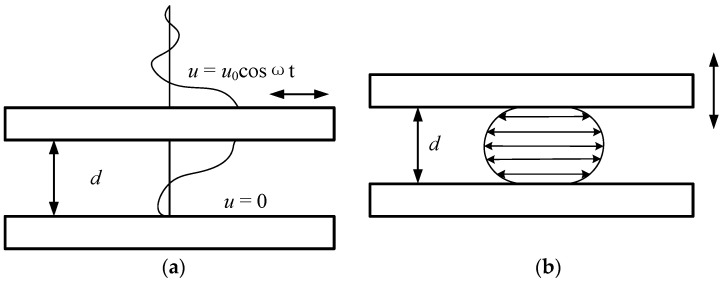
Viscous damping model: (**a**) slide film damping model; (**b**) squeezing film damping model [28].

**Figure 5:**
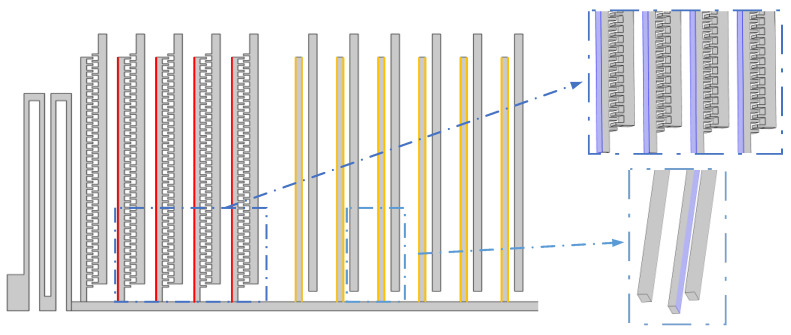
Schematic diagram of the squeeze film damping distribution in the 1/4 solution domain: red and yellow lines indicate squeeze film damping between the sidewalls of the back of the main arm of the driving electrode and between the shutters and the sensing strips, respectively.

As shown in Figure 4a, when the plate capacitor structure moves in parallel, the air between the two plates hinders the movement of the plate due to the action of the viscous force, resulting in synovial damping. As shown in Figure 4b, the squeeze film damping is caused by the relative movement of two parallel plates that squeeze the gas film between the plates. When two parallel plates are close to each other, the viscous force generated by the squeezing hinders the movement of the plates and causes them to lose energy. When the two parallel plates move away from each other, the viscous pulling force generated by the flowing gas is applied to the plates as a dissipation force. The calculation example in this article mainly includes slide film damping between the movable part and the substrate, squeeze film damping between the sidewalls of the back of the main arm of the driving electrode (indicated by the red lines), and squeeze film damping between the shutters and the sensing strips (indicated by the yellow lines), which is shown in detail in Figure 5.

**Figure 6:**
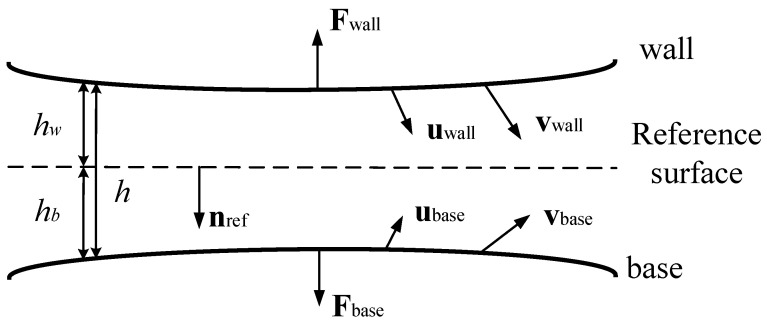
Schematic diagram of the thin film flow interface [29].

The mentioned viscous damping belongs to the flow in the gap (small normal direction and large tangential direction), whose viscous force is dominant, ignoring the inertial force. Therefore, the flow equation is established in the tangential direction, regardless of the normal pressure difference. Based on the above simplification, a simulation model is established. A schematic diagram of the model is shown in Figure 6.

The thin film fluid in the gap has two surfaces: the wall and the base, which correspond to the lower surface of the movable structure and the upper surface of the substrate, the sidewalls of shutters and the adjacent sidewall of the sensing electrodes, and the sidewalls of the main arm of the movable driving electrode and the sidewalls of the adjacent fixed driving electrode. The fluid domain is in the middle. The displacement and velocity data of the walls of the movable structure are directly derived from the solid field, which means that this part of the air damping is directly coupled with three fields.

This model uses the following modified Reynolds equation to solve:

(12) $$p_{\mathrm{tot}}(v_{b}\cdot n_{r}-v_{w}\cdot n_{r})+i\omega h_{0}p_{f}+\nabla_{t}\cdot (h_{0}p_{\mathrm{tot}}v_{\mathrm{av}})\;-\;p_{\mathrm{tot}}(v_{w}\cdot \nabla_{t}h_{w}+v_{b}\cdot \nabla_{t}h_{b})=0,$$

(13) $$v_{\mathrm{av}}=\frac{1}{2}(I-n_{r}n_{r}{}^{T})(v_{w}+v_{b})-\frac{h_{0}^{2}}{12\mu }\nabla_{t}p_{f},$$

where $v_{w}$ and $v_{b}$ are the velocity of the wall and base, $v_{\mathrm{av}}$ is the average value of the film velocity perpendicular to the surface at a point on the reference surface, $n_{r}$ is the normal vector perpendicular to the reference surface, $h_{0}$ is the average height of the membrane, $h_{w}$ and $h_{b}$ are the distance from the wall and base to reference surface, μ is dynamic viscosity, $p_{f}$ is the pressure generated by the flow, and $p_{\mathrm{tot}}$ is the total pressure ($p_{\mathrm{tot}}=p_{A}+p_{f}$, where $p_{A}$ is the ambient pressure).

#### 2.2.2. Incompressible Flow Damping

The air damping caused by the movement of the movable comb teeth relative to the fixed comb teeth and back-and-forth movement motion of the folded-flexure suspension in the microresonator, whose fluid characteristic structure and boundary conditions are more general, cannot be modeled simply using the aforementioned squeeze film damping and slide film damping. In terms of a MEMS system operating under atmospheric pressure, fluid can be regarded as incompressible because the density of air changes very little. Based on the above simplification, a numerical model based on Navier–Stokes equations is used:

(14) ∇⋅u=0,

(15) $$\rho \frac{\partial u}{\partial t}+\rho u\cdot \nabla (u)=-\nabla p+\nabla \cdot (\mu (\nabla u+{\left(\nabla u\right)}^{T}))+F,$$

where u is the fluid velocity, p is the fluid pressure, ρ is the fluid density, and F is the external force acting on the fluid.

**Figure 7:**
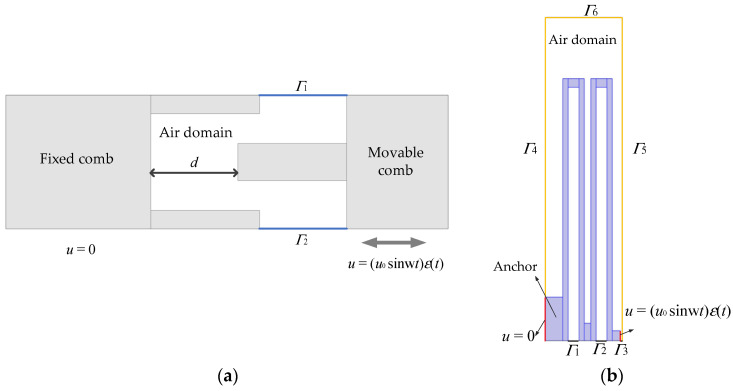
Incompressible fluid damping model: (**a**) 2D schematic diagram of the fluid-solid coupling model of the comb tooth unit; (**b**) 2D schematic diagram of the fluid-solid coupling model of the folded-flexure suspension.

A simulation model is then established to calculate the viscous damping between the interdigitated comb teeth and the air damping of the sidewalls of the folded beam. Because incompressible flow damping has a linear relationship with the number of comb teeth, a comb unit model is established for simplification, as shown in Figure 7a, and the complete viscous damping of the comb teeth is obtained by multiplying by the number of comb teeth. The movable comb teeth are set to specify the body displacement movement with a sine function, and the fixed comb teeth remain stationary. In the air domain, except for the upper and lower sides (shown by the blue line in Figure 7a, which are symmetrical boundary conditions, others (upper and lower surfaces) are open boundaries. Similarly, a partial fluid-structure coupling model of the folded-flexure suspension is established, as shown in Figure 7b. The rightmost side of the folded-flexure suspension is set to specify the displacement movement with a sine function, and a fixed constraint is set on the leftmost side of the anchor. To ensure the convergence of the solution, a smooth step function that is second order continuous is added to the sine function.

### 2.3. Electromechanical Coupling Calculation Model

To facilitate the calculation, the above model is reasonably simplified as follows: (1) Assume that the movement displacement-time curve of the micro-comb resonator is in the form of a sine function; (2) The micro-resonator is a differential drive structure, so 1/2 of the structure is taken as calculation domain; (3) The structural layer is processed from single crystal silicon, assuming that it is a linear elastic material.

**Figure 8:**
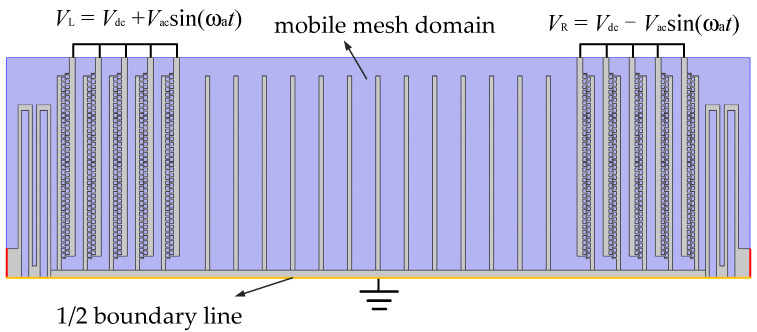
Schematic diagram of the electromechanical coupling model of the MEMS electric field sensor.

A three-dimensional model of the MEMS electric field sensor is established, as shown in Figure 8. The model has three domains: an electrostatic domain, a structural mechanics domain, and a mobile mesh domain. In the electrostatic solution domain, the static comb teeth at both ends are applied with DC bias and reverse AC voltage, and the movable structure is grounded; in the mobile mesh solution domain, all air gaps are selected as the deformation domain, which automatically introduces gap shrinkage due to structural deformation, and the air boundary on the 1/2 boundary line is a symmetric boundary condition; in the structural mechanic’s solution domain, the anchors and static combs at both ends are set as fixed constraints, and the 1/2 boundary line is the symmetric boundary condition.

Regarding the application of damping, as mentioned earlier, for slide film damping and squeeze film damping of simple double-layer flat structures, the thin film flow interface can be used for direct coupling. For incompressible flow damping of irregular structures (comb elements and folded-flexure suspension), it is necessary to extract the damping coefficient in the fluid-structure coupling time domain model. Since the damping of the folded-flexure suspension is independent of the movement displacement, it can be directly substituted as a constant. The damping coefficient of the comb teeth changes with the distance (*d*) between the movable comb teeth and the fixed comb teeth in the time domain. When the distance between the comb teeth decreases, the air film in the middle is squeezed, which makes the damping coefficient larger. As described in Section 2.2, to avoid the direct coupling of the three fields, the fitting function of the damping coefficient with the movement displacement (*x*) is extracted and converted into a frequency domain expression according to equal energy loss. Therefore, the correlation function between damping and amplitude is used as the feedback adjustment of the electromechanical coupling model so that the solution result can be adjusted automatically. The distracted curve between damping and amplitude value is the indirect coupling relationship between the displacement field and the flow field. The electrostatic-fluid-structure three-field indirect coupling can be realized by repeated iterations.

## 3. The Numerical Calculation Results of the Coupled Field

Based on the above model, the coupled solution is performed in COMSOL Multiphysics software, and the calculation results of the fluid load, vibration mode, and displacement field of the microresonator are obtained. Technical data about the main physical and mechanical properties of the material, the mesh type, analysis type, pressure, temperature are shown in Table 2. In addition, to verify the accuracy of the simulation, a vibration experiment was designed for comparison and verification.

### 3.1. Fluid Load

**Figure 9:**
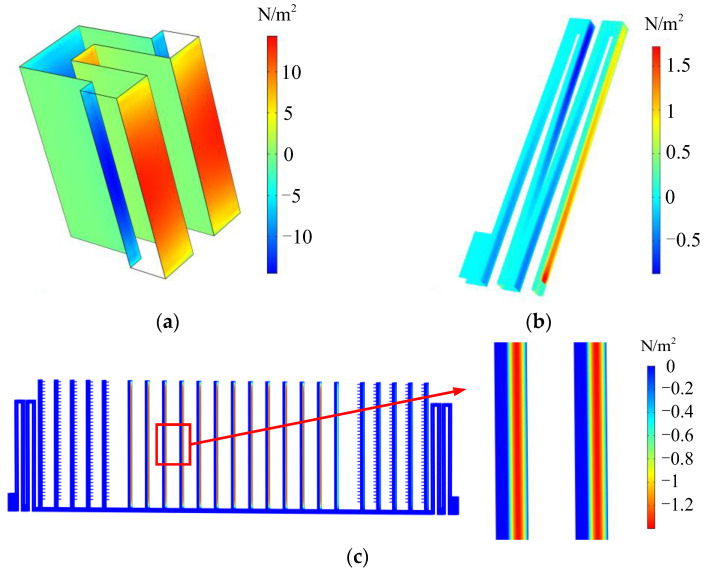
The fluid load distribution: (**a**) incompressible flow load distribution of the comb tooth unit; (**b**) incompressible flow load distribution of the folded-flexure suspension; (**c**) viscous flow load distribution of the shutters.

Based on the above model, the fluid-structure coupling time domain solution was carried out. The fluid load distribution of the comb-tooth unit, the folded-flexure suspension, and the shutters are shown in Figure 9.

It can be seen in the figure that the distribution of the fluid load distribution along the thickness direction is high in the middle and low on both sides. The viscous damping forces are obtained by surface integration of the fluid load of each part of the resonator. The damping coefficient is obtained by dividing the viscous damping force by the vibration speed. The simulation results of the damping coefficient at the equilibrium position at resonance are shown in Table 3. In the table, the slide film damping of the sensor is much smaller than the squeezing film damping of each part. The viscous damping of the sensor operated at atmospheric pressure and an ambient temperature of 293.15 K is mainly composed of the incompressible flow damping of the comb teeth (including both the sliding film and the squeezing film damping), which is an order of magnitude larger than those of other parts.

**Figure 10:**
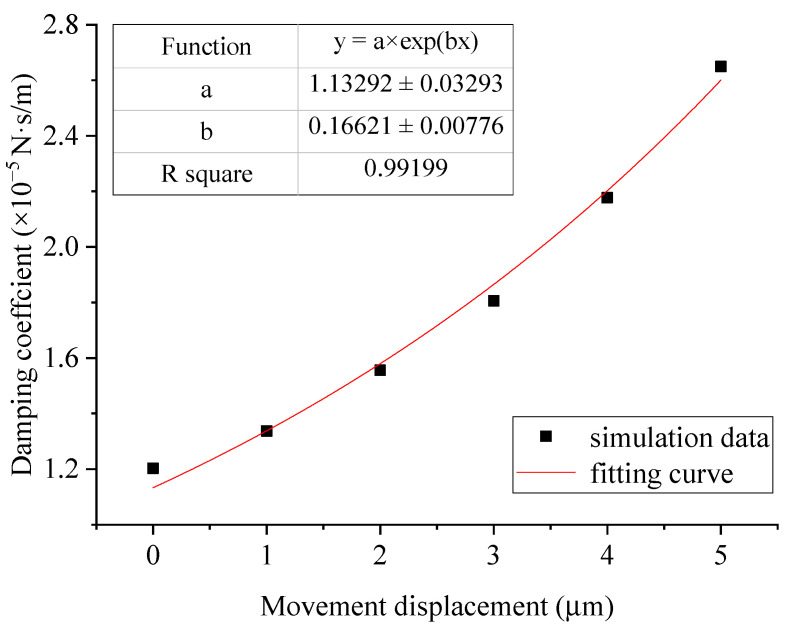
Variation in comb tooth damping with movement displacement and its fitting curve.

For the varying damping of the comb-tooth unit, the simulation values of the damping coefficient under different movement displacements *x* and the fitting function of the two are shown in Figure 10.

### 3.2. Vibration Mode and Displacement Field of the Microresonator

**Figure 11:**
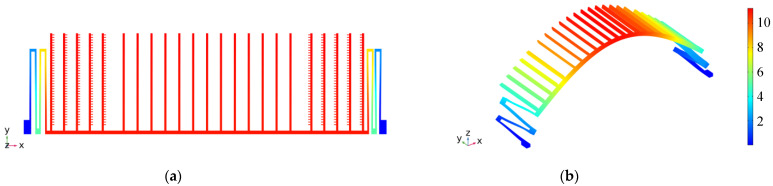

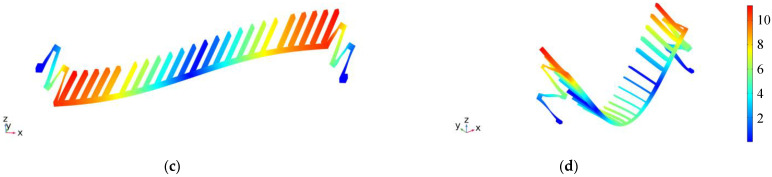
The first four-order eigenmodes deformation diagram of the MEMS microstructure: (**a**) First-order: parallel X-direction vibration mode; (**b**) Second-order: Z-direction vibration mode; (**c**) Third-order: Y-axis as rotation axis, small-amplitude flip and swing in XZ plane; (**d**) Fourth order: the flip vibration mode in the Z direction. The numerical value on the color legend represents the relative value of the mode shape.

**Figure 12:**
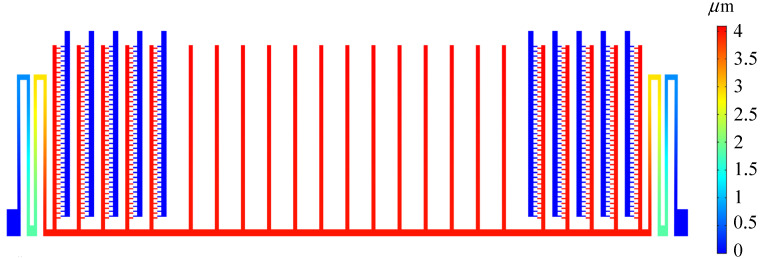
Displacement field distribution of the MEMS sensor.

Based on the above model, the resonant frequency and displacement amplitude are obtained by the solution of coupling domains. Figure 11 shows a set of deformation diagrams of the first four vibration eigenmodes of the MEMS microstructures. A vibration system can be expressed as the linear superposition of infinite-order eigenmodes. The first-order is the wanted vibration form. Table 4 shows the characteristic frequencies corresponding to the first four modes of the MEMS sensor. It can be seen in the table that the characteristic frequencies of the higher-order eigenmode are far from that of the first-order eigenmode, which meets the design requirements. Figure 12 demonstrates the displacement field distribution of the MEMS sensor at a DC bias voltage of 29.7 V and an AC voltage of 2.7 V. Under this excitation voltage, the resonance amplitude of the movable structure reaches 4.3 µm.

## 4. Calculation Accuracy Verification

### 4.1. Experimental Comparison

To verify the accuracy of the calculation results, a comparative experiment was carried out. A driving voltage was applied to the actual microelectrostatic resonator according to the circuit diagram of Figure 1, namely:

(16) $$V_{L}=V_{\mathrm{dc}}+V_{\mathrm{ac}}\sin\omega t$$

(17) $$V_{R}=V_{\mathrm{dc}}-V_{\mathrm{ac}}\sin\omega t$$

**Figure 13:**
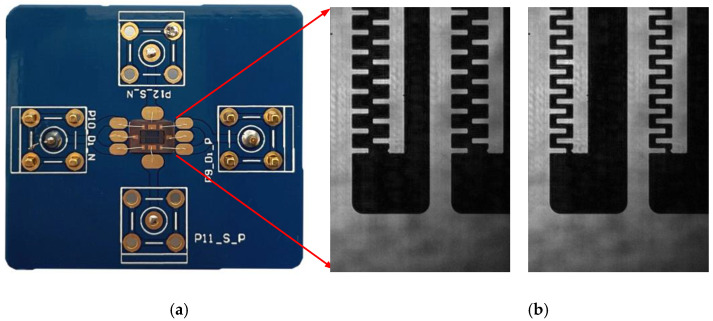
MEMS electrostatic comb resonator images: (**a**) PCB image; (**b**) SEM images of different movement displacement.

Vibration tests were performed at atmospheric pressure and at an ambient temperature of 298.15 K with a Lyncee Tec DHM R2104 digital holographic microscope. The PCB image and SEM images of different movement displacements of the MEMS electrostatic comb resonator are shown in Figure 13.

Due to the softening effect of DC voltage, the natural frequency of the resonant system changes with DC bias voltage. In the experiment, the MEMS resonator was analyzed by sweeping frequency with a step of 1 Hz under three different sets of DC bias ($V_{dc}=15,18,21V$). Then AC excitation of different amplitudes is applied at the measured resonance frequencies, respectively. The vibration displacement data were processed to obtain the measured resonance amplitudes. In contrast, the simulation data of the resonance frequency under the same voltage excitation and the displacement amplitudes under the resonance frequencies are calculated.

**Figure 14:**
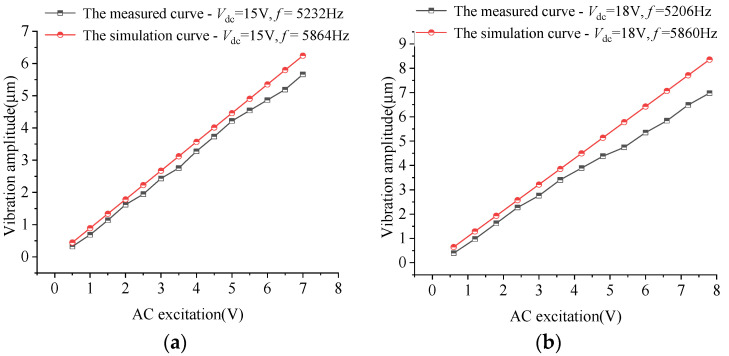

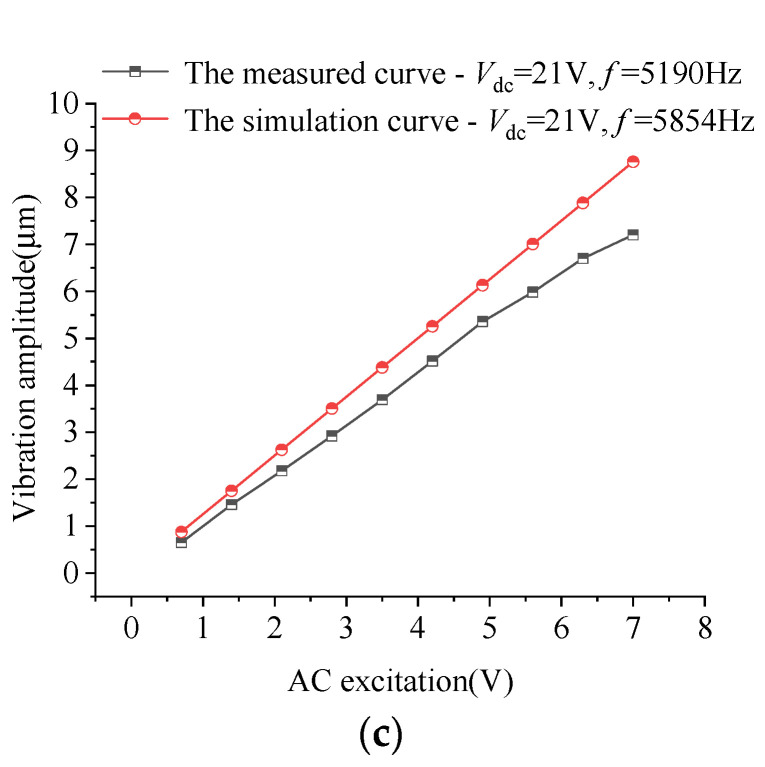
Comparison curves of the measured vibration amplitude of the MEMS sensor and the simulated value: (**a**)*V*_dc_ = 15 V; (**b**)*V*_dc_ = 18 V; (**c**)*V*_dc_ = 21 V.

Figure 14 shows the comparison curves of the measured vibration amplitudes and the simulated value of the MEMS sensor under three different sets of DC bias. It can be seen in the figure that the average errors of the resonance frequencies and vibration amplitudes are 12.48% and 15.47%, respectively. To take into account the measurement accuracy and avoid the relative error divided by a smaller number, the points with a vibration amplitude less than 1 μm are not included. The simulated displacement amplitude is slightly higher than the actual amplitude. The error of simulation and actual measurement comes from neglected damping, process error (accuracy tolerance is 1 µm), measurement error, error caused by equivalent linearization. Among them, the neglected damping includes internal losses in the material, friction in joints, sound emission, anchor losses, and thermoelastic damping. In addition, the warping of the cantilever beam is observed in the manufacturing of the sensor, and the height difference is up to 0.5–2 µm, which is not considered in the simulated model. The experimental results confirm that the calculated results in this paper are consistent with the measured values within the allowable error range and have a higher accuracy than that of the finite element dynamic simulation of the general micromechanical system (50–80%).

### 4.2. Influence of Damping Nonlinear Processing on Simulation Accuracy

To compare the improvement of simulation accuracy by damping nonlinear processing, three different types of microelectrostatic comb resonators (see Table 5 for specific parameters) were selected for comparison. The nonlinearity of air damping is inversely proportional to the gap between comb teeth and proportional to the thickness of the structure layer. Therefore, under the same vibration amplitude, Type 3 damping and its rate of change are greater than those of Type 2 and Type 1.

**Figure 15:**
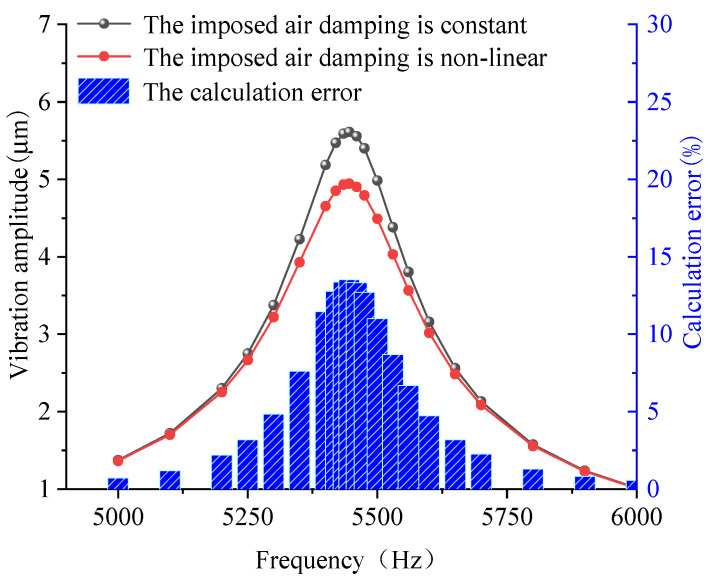
The frequency sweep simulation curves of the Type 3 resonator with or without damping nonlinear processing and the error percentages.

Figure 15 shows the frequency sweep simulation curves of the Type 3 resonator with or without damping nonlinear processing and the error percentages. Among them, the damping linear processing group takes the damping as the constant damping. Figure 15 shows that for a vibration system with significant nonlinearity, the calculation error caused by taking the air damping as a constant is relatively large, reaching 13.5%. In addition, the amplitude error in the calculation of resonance is maximized.

Figure 10 in Section 3.1, where the comb-tooth damping changes with the movement displacement shows that the nonlinearity of the damping is related to the slope of the function. Therefore, the damping nonlinear strength of the mechanical vibration system (*S*_nonlinear_) is set as the damping difference between the resonator amplitude and the equilibrium point divided by the vibration amplitude, namely:

(18) $$S_{\mathrm{nonlinear}}=\frac{{c|}_{x=\mathrm{Amplitude}}-{c|}_{x=0}}{\mathrm{Amplitude}}$$

**Figure 16:**
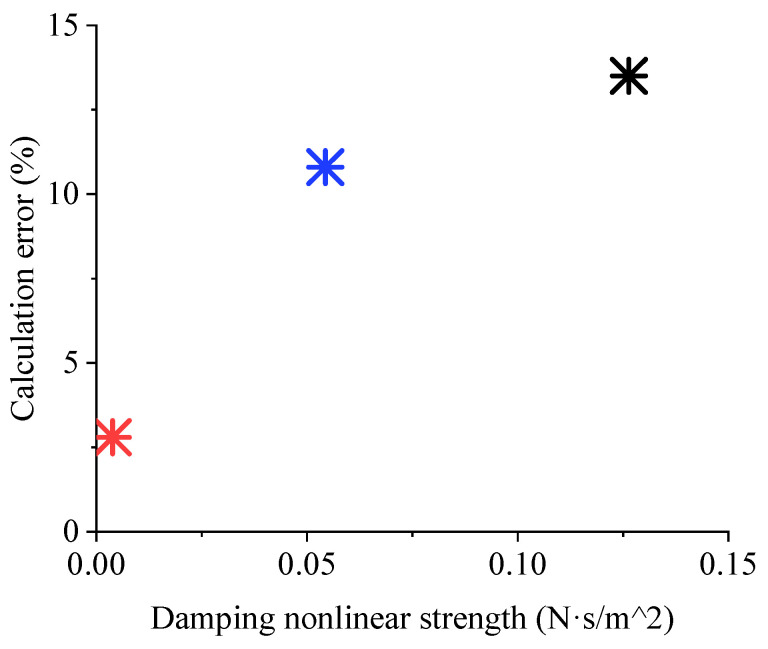
The relationship between the nonlinear strength of the three types and the improvement of simulation accuracy by damping nonlinear processing. The red, blue and black * denote Type 1, 2 and 3, respectively.

Figure 16 shows the relationship between the nonlinear strength of the three types at their respective vibration amplitudes and the improvement of simulation accuracy by damping nonlinear processing. The greater the nonlinearity of the system is, the greater the error caused by linearization. Therefore, in a system with significant damping nonlinearity, especially when calculating the amplitude at resonance, the nonlinear characteristics of damping must be considered.

## 5. Conclusions

In this paper, we propose a finite element simulation model of the mechanical vibration of a MEMS electrostatic comb resonator, which uses COMSOL software as a solution tool. The key contribution of this paper is the novel electrostatic-fluid-structure three-field indirect coupling method to avoid the computational complexity and uncertainty of the results of three-field direct coupling. Compared with electromechanical coupling model with a constant air damping coefficient, this approach can comprehensively consider the influence of nonlinear damping concerning the large displacement of the structure, and the microscale effect to significantly improve the accuracy of the simulation. Compared with the measured data, it can be concluded that the amplitude and resonance frequency of the microresonator considering the nonlinear damping force are very close to the experimental values (amplitude average error: 15.47%, resonance frequency error: 12.48%). This article provides a reference for the analysis and optimization design of a class of electrostatically driven MEMS devices, such as microresonators, micromirrors, and micropumps.

We also propose a block modeling method for the viscous damping of a microresonator. To model the air damping of the plate structure, sliding film damping and squeezing film damping are used to realize direct coupling. For movable parts with a general fluid characteristic structure and boundary conditions, a fluid-structure coupling time-domain analysis model is established to extract the fitting curve of the damping coefficient with the displacement of the motion. The time-frequency domain conversion of viscous damping is performed through a proposed method in which the energy loss in air damping per unit period is equal. As the previous theoretical derivation can be obtained, its essence is equivalent linearization, and its error term is the high-order term of the Fourier expansion. In addition, by comparing the impact of nonlinear damping processing on the simulation accuracy, it can be seen that in a system with significant damping nonlinearity, especially when calculating the amplitude under resonance, the nonlinear characteristics of damping must be considered.

## Figures and Tables

**Table 1:** Main parameters of Type 1 MEMS resonator.

Parameter	Type 1
Thickness of top silicon film—*h* (μm)	30
Suspension beam length—*l* (μm)	600
Suspension beam width—*w* (μm)	12
Number of drive combs (each side)	36
Drive comb gap—*g* (μm)	12
Drive comb distance—*d* (μm)	4
Material	Monocrystalline silicon

**Table 2:** Technical data of simulation model.

Components	Category	Technical Data
Material	Young’s modulus	190 GPa
	Poisson’s ratio	0.28
Environmental parameters	Temperature	293.15 K
	Pressure	1.0133 × 10^5^ Pa
Meshing	Mesh type	Free triangular mesh + swept mesh
	Degrees of freedom	1,162,486
Fluid–-solid coupling	Analysis type	Transient analysis
Electromechanical coupling	Analysis type	Frequency domain analysis

**Table 3:** Calculation results of viscous damping at resonance.

Damping Type	Model	Damping Coefficient c (N·s/m)
Relative movement between comb teeth	Incompressible flow damping	$2.268\times 10^{-5}$
Sides of the folded-flexure suspension	Incompressible flow damping	$2.033\times 10^{-6}$
Dorsal side of main comb arm	Squeeze film damping	$2.634\times 10^{-7}$
Sides of the shutter	Squeeze film damping	$2.536\times 10^{-6}$
Surface	Slide film damping	$4.576\times 10^{-8}$

**Table 4:** The characteristic frequencies corresponding to the first four modes of the MEMS sensor.

Eigenmodes	Characteristic Frequencies (Hz)
First-order	5739.7
Second-order	7930.7
Third-order	18,258.4
Fourth-order	30,015.3

**Table 5:** Simulation modeling parameter table of MEMS resonators.

Parameter	Type 1	Type 2	Type 3
Film thickness (μm)	30	30	40
Drive comb gap (μm)	12	6	6

## Data Availability

Not applicable.
